# Microalgae-Templated Spray Drying for Hierarchical and Porous Fe_3_O_4_/C Composite Microspheres as Li-ion Battery Anode Materials

**DOI:** 10.3390/nano10102074

**Published:** 2020-10-20

**Authors:** Jinseok Park, Jungmin Kim, Dae Soo Jung, Isheunesu Phiri, Hyeon-Su Bae, Jinseok Hong, Sojin Kim, Young-Gi Lee, Myung-Hyun Ryou, Kyubock Lee

**Affiliations:** 1 Department of Chemical and Biomolecular Engineering, Korea Advanced Institute of Science and Technology (KAIST), 291 Daehak-ro, Yuseong-gu, Daejeon 34141, Korea; jspark80@seas.upenn.edu; 2 Department of Chemical and Biological Engineering, Hanbat National University, 125 Dongseo-daero, Yuseong-gu, Daejeon 34158, Korea; bigjlove77@gmail.com (J.K.); isheunesuphiri@gmail.com (I.P.); hsbae1234@gmail.com (H.-S.B.); sazza9703@gmail.com (J.H.); sojinn1216@gmail.com (S.K.); 3 Energy and Environmental Division, Korea Institute of Ceramic Engineering and Technology, 101 Soho-ro, Jinju 52851, Korea; dsjung@kicet.re.kr; 4 Intelligent Sensors Research Section, Electronics and Telecommunications Research Institute (ETRI), Daejeon 34129, Korea; 5 Graduate School of Energy Science and Technology, Chungnam National University, Daejeon 34134, Korea

**Keywords:** spray drying, microalgae, Fe_3_O_4_/C composite microsphere, hierarchical pore, Li-ion battery, anode

## Abstract

A method of microalgae-templated spray drying to develop hierarchical porous Fe_3_O_4_/C composite microspheres as anode materials for Li-ion batteries was developed. During the spray-drying process, individual microalgae serve as building blocks of raspberry-like hollow microspheres via self-assembly. In the present study, microalgae-derived carbon matrices, naturally doped heteroatoms, and hierarchical porous structural features synergistically contributed to the high electrochemical performance of the Fe_3_O_4_/C composite microspheres, enabling a discharge capacity of 1375 mA·h·g^−1^ after 700 cycles at a current density of 1 A/g. Notably, the microalgal frameworks of the Fe_3_O_4_/C composite microspheres were maintained over the course of charge/discharge cycling, thus demonstrating the structural stability of the composite microspheres against pulverization. In contrast, the sample fabricated without microalgal templating showed significant capacity drops (up to ~40% of initial capacity) during the early cycles. Clearly, templating of microalgae endows anode materials with superior cycling stability.

## 1. Introduction

Li-ion batteries (LIBs) have been widely applied to portable electronic devices, even while ongoing challenges such as development of a reliable and large-scale energy storage system remain. To overcome the limited energy density of conventional anode material such as graphite (372 mA·h·g^−1^), alternative anode materials for LIBs, including Si and a variety of metal oxides (MO_x_; *M* = Cr, Mn, Fe, Co, and Ni), have been proposed [1,2]. In particular, Fe_3_O_4_ has drawn great attention for its potential application to anode materials, given its high theoretical energy density (928 mA·h·g^−1^), nontoxicity, abundance, and low cost [3,4]. However, like other metal oxides and Si, it suffers from large volume changes during charge/discharge reactions. Significant volume change leads to repeated exposure of active materials to electrolytes and consequent unstable growth of the solid electrolyte interphase (SEI) on the active materials. Resultantly thickened SEI layers drastically impede the transportation of Li ions, thereby incurring failure of electrochemical cells. Indeed, the stable formation of SEI layers is essential in order to achieve satisfactory electrochemical performance of anode material, especially considering the low electrical conductivity of pristine iron oxide [5,6].

To tackle these problems, various strategies have been proposed, such as controlling the sizes, shapes, and structures of iron-oxide-based anode materials or fabricating iron oxide/carbon composite materials [7,8,9,10,11,12,13,14]. Nanostructured active materials often exhibit improved electrochemical performance due to the large surface area available for contact with the electrolytes, the short diffusion length of Li ions, and the enhanced mechanical stability against pulverization [1,6,11,12,13]. In addition, the hollow and porous morphologies of nanostructured materials are beneficial to accommodation of the volume change associated with Li ion intercalation [13,14,15,16,17]. Hybridization of active materials including iron oxide with carbon has been introduced as another means of buffering volume expansion, resulting in enhanced electrochemical performance with improved structural stability [7,8,9,10].

In light of these considerations, biotemplating techniques have been explored, because various living organisms provide an opportunity to design composite materials of favorable structure. For example, banana peels, bacteria, and green leaf have been used for hierarchical porous structures, hollow spheres, and morph structures, respectively [18,19,20]. Moreover, microorganisms have been extensively utilized in the fabrication of carbon composite materials, and have shown the benefits of introducing them as a carbon template [21,22]. It is well known that the functional groups on cell walls enable facile adsorption and assembly of the inorganic elements for fabrication of carbon composite materials [23]. In fact, we recently showed that lipid-extracted oleaginous microalgae could be utilized for fabrication of nano- and micro-structured composite anode materials of LIBs; and indeed, this is a promising means of recycling microalgal biomass after obtaining lipid, the main target material for biodiesel production [19,20].

In this study, hierarchical porous Fe_3_O_4_/C composite microspheres were developed as LIB anode materials by using a spray-drying method with lipid-extracted microalgae as carbon templates. The unique raspberry-like Fe_3_O_4_/C composite microspheres with hierarchical pores were formed through the self-assembly of building blocks of lipid-extracted microalgae. In the formation process, the hollow structure, the first level of the pore, is generated by surface precipitation of microalgae from aerosol droplets, while the second level of macropores is occupied by the individual microalgae. The third level of mesopores is established through carbonization of microalgae. This hierarchical porous structure is beneficial for cyclability with improved accommodation of volume changes. In addition, in the present study, naturally and homogeneously doped phosphorus and sulfur ions from the microalgae were identified in the composite materials, which ions can help to enhance electrochemical performance. These features enabled the electrochemical performance of Fe_3_O_4_/C composite microspheres as anode materials of Li-ion batteries (LIBs): a high capacity of ~1300 mA·h·g^−1^ up to 700 cycles at a current density of 1 A·g^−1^. The microalgal frameworks of the Fe_3_O_4_/C composite microspheres’ hierarchical and porous structure provide for both structural stability and higher electrochemical performance.

## 2. Materials and Methods

### 2.1. Preparation of Lipid-Extracted Microalgae

*Chlorella* sp. KR-1 was used in this experiment. Its cultivation conditions are described in reference [24]. After separating microalgae by centrifuging the culture medium at 5000 rpm for 15 min, lipid was extracted by using a mixed organic solution of chloroform (CHCl_3_, purity = 99%, Junsei Chemical, Tokyo, Japan) and methanol (CH_3_OH, purity = 99.8%, Junsei Chemical, Tokyo, Japan) at a volume ratio of 1:1. This organic solution allows the extraction of both fat-soluble and water-soluble lipid by chloroform and methanol, respectively. The amount of chloroform/methanol solution was 100 mL for 5 g of centrifuged microalgae (wet), and this mixture had been vigorously stirred at 800 rpm for 20 h for complete lipid extraction. Residual microalgae were separated from the chloroform/methanol solution and washed with deionized water 3 times.

### 2.2. Synthesis of Fe_3_O_4_/C Composite Microspheres

Lipid-extracted microalgae were dispersed in 0.16 M of iron nitrate nonahydrate (Fe(NO_3_)_3_∙9H_2_O, 97%, Sigma Aldrich, St. Louis, MO) aqueous solution. This solution was spray-dried by using a mini spray dryer (B-290, BUCHI, Essen, Germany) with a feeding rate of 3 mL·min^−1^ and an inlet temperature of 180 °C with air as a carrier gas. The outlet temperature was maintained between about 115 and 120 °C by regulating the aspirator. Spray-dried samples were further annealed in two steps: first, pre-annealing at 300 °C in an air atmosphere for 1 h at a heating rate of 5 °C·min^−1^ followed by cooling; second, post-treatment at 500 °C in a nitrogen atmosphere for 1 h with the same heating and cooling procedure. For comparison, 0.16 M of iron nitrate solution was spray-dried without microalgal templates, and the samples thus obtained were heat-treated identically.

### 2.3. Characterization

The morphology and crystal structure of the composite materials were investigated by field-emission scanning electron microscopy (SEM, Magellan 400, FEI, Hillsboro, OR, USA) and X-ray diffraction (XRD, D/MAX-2500, RIGAKU, Tokyo, Japan), respectively. The nitrogen gas adsorption–desorption measurements were performed using a ASAP2420 (Micromeritics, Nocross, GA, USA) at 77K and 10^−5^–0.99 of relative pressure range (P/P_0_). The specific surface area and pore size distribution of the samples were determined by the Brunauer-Emmett-Teller (BET) method and Barrett-Joyner-Halenda (BJH) methods, respectively. The micropore size distribution was evaluated by Horvath-Kawazoe (H-K) method. Thermogravimetric analysis (TGA, TG 209 F3, NETZSCH, Selb, Germany) was carried out under an air atmosphere to identify the remaining contents of carbonaceous materials in the composite microspheres. X-ray photoelectron spectroscopy (XPS, Sigma Probe, Thermo VG Scientific, Waltham, MA, USA) was used to determine the surface’s elemental composition. The cross-sectional morphology of the synthesized composite microspheres was observed with transmission electron microscopy/scanning transmission electron microscopy (TEM/STEM, Titan cubed G2 60-300, FEI, Hillsboro, OR, USA) after preparing a thin-layered specimen with a focused ion beam (FIB, Helios NanoLabTM, FEI, Hillsboro, OR, USA) at the National Nanofab Center (NNFC). Before the FIB procedure, the composite microspheres were molded in epoxy resin to minimize structural damage during the milling process. TEM observation of thin-layered composite anode materials was also conducted after electrochemical measurements (50 charge/discharge cycles).

### 2.4. Electrochemical Measurements

The electrochemical properties were determined using a 2032-type coin cell. The electrode was prepared by mixing 70 wt.% active material, 20 wt.% Super P, and 10 wt.% carboxymethyl cellulose (CMC) binder. The loading level of the electrode was ~0.36 mg·cm^–2^. Li metal and polyethylene film were used as the counter electrode and separator, respectively. Li hexafluorophosphate (LiPF_6_, 1.15 M) in a mixture of ethylene carbonate (EC)/dimethyl carbonate (DMC) (3/7, *v*/*v*, Enchem Co., Ltd., Chunan, Korea) was used as the electrolyte. The charge/discharge characteristics of the composite electrodes were determined by cycling between 0.01 and 3.0 V vs. Li/Li^+^ at a constant current density of 1 A·g^−1^.

## 3. Results and Discussion

**Figure 1:**
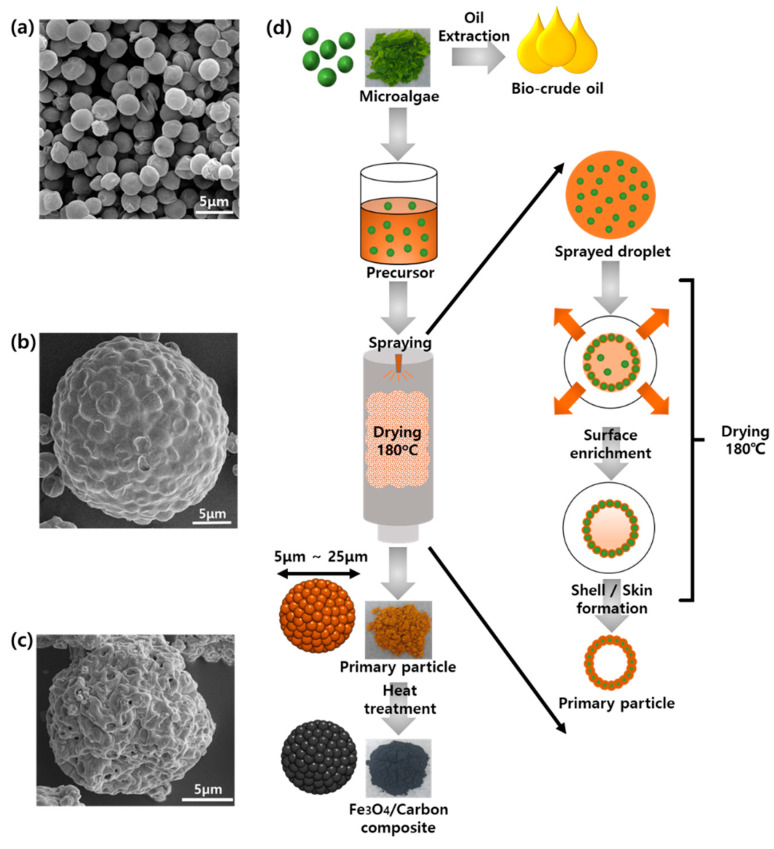
Scanning electron microscopy (SEM) images of (**a**) lipid-extracted microalgae, (**b**) spray-dried primary particles before annealing, and (**c**) Fe_3_O_4_/C composite microspheres after annealing. (**d**) Schematics of spray drying procedure and development of shell/skin formation during drying process.

Figure 1 presents a schematic illustration in the form of SEM images of the process of synthesizing Fe_3_O_4_/C composite microspheres from the harvested microalgae. First, lipid-extracted microalgae (2–3 μm in diameter, Figure 1a) were mixed with an iron nitrate solution. The solution was spray-dried to form the self-assembled and hierarchical microspheres (5–25 μm in diameter, Figure 1b). As indicated in Figure 1d, when the atomized droplets enter the drying chamber at 180 °C, necessitated evaporation of solvent (water) leads to the surface enrichment of the microalgae and iron component owing to the thermal gradient. Then, precipitation starts from the surface, and microalgal building blocks are self-assembled into the hollow spherical structure (the first level of pores) upon the completion of the drying process. The hollow and spherical particles can be synthesized by spray drying of colloidal suspension with colloids as templates, microalgae in this case [25,26]. Figure 1b shows the raspberry-like morphology of the spray-dried primary particles, which were obtained by the self-assembly of microalgae into hierarchical microspheres. Note that the surface of the primary particle has no apparent porous structure, because iron nitrate components fill the gap between the relatively large microalgae when they are assembled.

The spray-dried particles were further annealed to Fe_3_O_4_/C composite microspheres through two sequential heat treatments: 300 °C in an air atmosphere for 1 h followed by 500 °C in a nitrogen atmosphere for 1 h. It is necessary to anneal the sample at 500 °C in order to fully decompose the iron nitrate precursor. Just one-step heat treatment at 500 °C in air results in the oxidation of all carbon contents, whereas just one-step heat treatment at 500 °C under nitrogen conditions produces electrochemically inactive Fe crystal due to the carbothermic reduction. Therefore, the two-step heat treatments were conducted, namely pre-annealing at 300 °C in air to decrease carbon contents and prevent over-reduction at 500 °C in nitrogen, which maintained ~15 wt.% of carbon contents (Figure S1 in Supplementary Materials) with the electrochemically active Fe_3_O_4_ phase. As shown in Figure 1c, the annealed composite microspheres exhibited wrinkled and porous structures while maintaining their hierarchical and spherical morphologies. The formation of the wrinkles and pores was attributed to the generation of CO_2_ as carbonaceous materials were oxidized during annealing under the air atmosphere. To evaluate the surface area and pore size distribution of the annealed composite microspheres, BET, BJH, and H-K theories were applied (Figure S2 in Supplementary Materials). The hysteresis in the adsorption–desorption isotherm indicates the presence of the mesoporous structure (the third level of pores) with a bit of micropores in the composite microspheres, with the pore sizes distributed within the mesopore range [27]. At a low P/P_0_ range of adsorption isotherm, BET analysis gives surface area of 17.2 m^2^·g^−1^. The mesoporous structure can enhance the electrochemical performance of composite anode materials by providing a short diffusion length for Li ions migration and a large surface area for direct contact with electrolytes. In addition, mesopores can effectively alleviate the volume change during the charge/discharge process, having the spatial room to accommodate the volume expansion and the synergetic effect with the surrounding carbon matrix [17,28,29].

**Figure 2:**
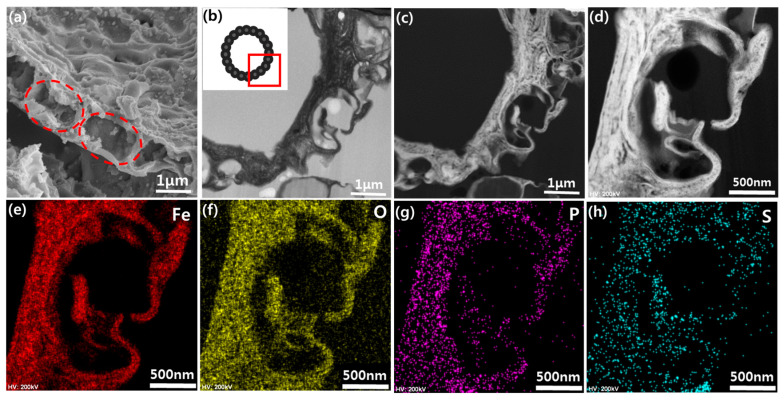
(**a**) Scanning electron microscopy (SEM) images of cross-section of Fe_3_O_4_/C composite microspheres. The red dotted circles highlight each templated each microalga. (**b**) Transmission electron microscopy (TEM) images of focused ion beam (FIB)-milled cross-section of Fe_3_O_4_/C composite with inset showing TEM-observed part. (**c**) Scanning transmission electron microscopy (STEM) image corresponding to Figure 2b. (**d**) Magnified STEM image of Figure 2c for energy-dispersive X-ray spectroscopy (EDS) mapping of (**e**) Fe, (**f**) O, (**g**) P, and (**h**) S.

To investigate the hollow structure of the spray-dried composite microspheres, the cross-sectional morphologies were identified by SEM (Figure 2a) and TEM (Figure 2b). First, the composite microspheres were intentionally crumbled to observe their cross-section by SEM. Figure 2a shows part of it, highlighting the area where the length scale is on the order of the size of microalgae, as indicated by the red circles. This demonstrates that the individual microalgae each serve as a building block for the formation of spherical and hollow structures. Notably, void spaces (the second level of pores) within the highlighted region originate from the lipid-extraction and annealing processes. Thus, composite microspheres consist of active materials of 100–200 nm thickness between every 2–3 μm sized void. These structural features are expected to effectively buffer the pulverization of active materials, since the extra space between the active materials can accommodate the volume change during cycling.

The cross-sectional morphology and elemental distribution of the composite microspheres were further investigated by focused ion beam (FIB) and TEM. Figure 2b represents the bright-field TEM image of the prepared specimen with the FIB cut, clearly showing the hollow structure of the composite microspheres with a similar size of void as observed in Figure 2a. The elemental distributions of the composite microspheres were identified by EDS mapping in the STEM mode. In Figure 2c–h, the dark background is an epoxy resin used for molding, and therefore the qualitative presence of carbon elements is not shown here, though the carbon contents had been determined by TGA to be ~15 wt.% (Figure S1 in Supplementary Materials). Figure 2e–h show that the Fe_3_O_4_/C composite microspheres contain Fe and O elements along with the homogeneously distributed P and S elements. This confirmed the presence of microalgae-driven P and S elements in the Fe_3_O_4_/C composites. These naturally doped ions are expected to enhance ionic conductivity during the charge/discharge process. In our previous work, N atoms were found in microalgae-templated composite microspheres, but they were absent from Fe_3_O_4_/C composite microspheres due to annealing in an air atmosphere [21,22].

**Figure 3:**
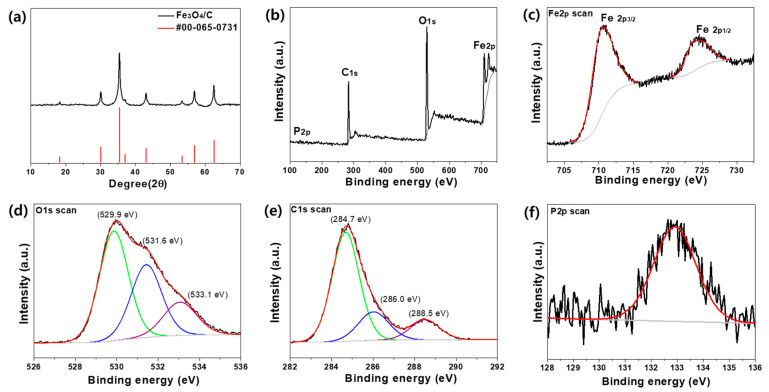
(**a**) XRD patterns of Fe_3_O_4_/C composite microspheres with JCPDS card #76-1849. XPS spectra of Fe_3_O_4_/C composite microspheres for (**b**) full scan, (**c**) Fe 2p, (**d**) O 1s, (**e**) C 1s, and (**f**) P 2p. In (**d**,**e**), the colored lines correspond to the given bonding energies. The red and gray lines are the enveloped and background signals, respectively.

In Figure 3a, the XRD pattern shows that the crystalline structure of the annealed composite microspheres is well-matched with the diffraction peaks of Fe_3_O_4_ (JCPDS card #76-1849). The well-defined peaks from the Fe_3_O_4_ lattice suggest a conversion of iron nitrate to electrochemically active iron oxide. The average crystallite size (*τ*) ~20.6 nm is obtained from the Scherrer equation, $\tau =\;\frac{K\lambda }{\beta cos\theta }$, where *K* = 0.9 for Scherrer constant, *λ* = 0.154 nm for wavelength of X-ray source (Cu Kα), *β* for full width at half maximum, and 2*θ* for the peak position. XPS further revealed the existing elements and their bonding characteristics in the composite microspheres. In Figure 3b, the XPS spectra of the composite microspheres exhibit clear peaks corresponding to the Fe 2p, O 1s, and C 1s peaks with the minority P 2p peak from the naturally doped P elements. The bonding characteristics of the S elements were not detected in the XPS analysis, due to the low concentration in the composite microspheres, which is qualitatively identified in Figure 2h. As shown in Figure 3c, the Fe peaks at 710.6 eV and 724.4 eV correspond to the spin-orbit coupling of Fe 2p_3/2_ and Fe 2p_1/2_, respectively [30]. Since γ-Fe_2_O_3_ has a distinguishable satellite peak at around 718.8 eV, no satellite signal between the two main peaks was the indication of Fe_3_O_4_ [30,31,32]. The binding characteristics of the O, C, and P elements in the Fe_3_O_4_/C composite microspheres are represented in Figure 2d–f, respectively. Among the deconvoluted peaks from O 1s, the strongest signal at 529.9 eV represents the oxygen elements in the Fe_3_O_4_ phase, while the others at 531.6 eV and 533.1 eV correspond to the O atoms bonded with C atoms in the composite microspheres. Accordingly, the C 1s spectra are comprised of three peaks at 284.7 eV, 286.0 eV, and 288.5 eV for the C–C, C–O, and C=O bonds, respectively [33,34]. The peak at 132.8 eV with relatively low intensity was found to be associated with the binding energy of P 2p_3/2_ in oxidized phosphorus, which suggests a presence of P atoms in the Fe_3_O_4_/C composite microspheres [35].

Figure S3 shows an SEM image of the obtained particulates from the spray drying of the iron nitration solutions without the microalgae. Even though the synthetic procedure was identical, it has no features of hierarchical and porous structures, due to the absence of microalgal templates. Instead, it is a bulky and shrunk sphere that is typically developed during the fast solvent evaporation of spray drying. The XRD patterns of the spray-dried and annealed samples without the microalgae correspond to α-Fe_2_O_3_, whereas the sample with microalgae had an Fe_3_O_4_ phase (Figure S4). The crystalline phase difference is attributable to the absence of carbon, which provides for a reduction atmosphere during heat treatment. Note that the theoretical capacity of α-Fe_2_O_3_ (1004 mA·h·g^−1^) is similar to that of Fe_3_O_4_ (928 mA·h·g^−1^).

**Figure 4:**
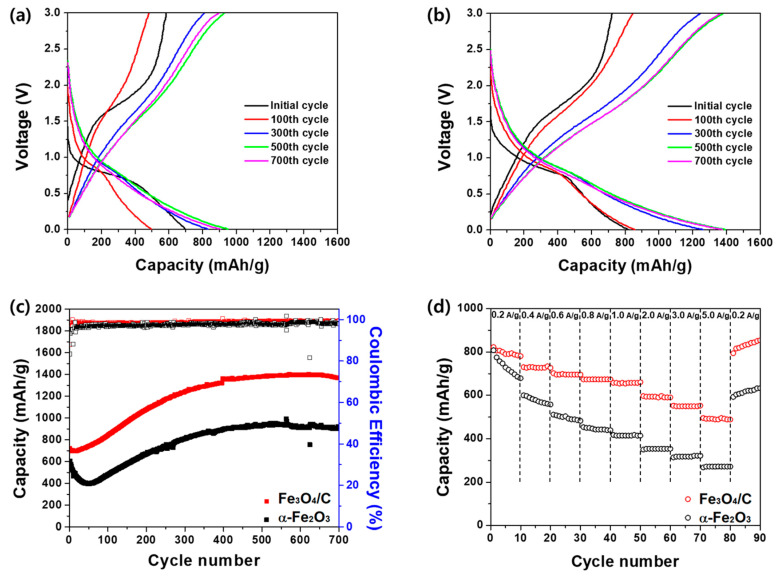
Electrochemical performances of α-Fe_2_O_3_ (without microalgae) and Fe_3_O_4_/C (with microalgae). Charge/discharge profiles of (**a**) α-Fe_2_O_3_ and (**b**) Fe_3_O_4_/C composite. (**c**) Cycling performance and coulombic efficiency of α-Fe_2_O_3_ and Fe_3_O_4_/C at current density of 1 A·g^−1^. (**d**) Rate capabilities of α-Fe_2_O_3_ and Fe_3_O_4_/C at varying current densities for every 10 cycles.

Figure 4a,b show the charge/discharge profiles of α-Fe_2_O_3_ (without microalgae) and the Fe_3_O_4_/C composite, respectively, at a current density of 1 A·g^−1^ in the voltage range of 0.01–3.0 V. In both α-Fe_2_O_3_ and Fe_3_O_4_/C, the voltage plateau at ~0.75 V during the first discharge corresponds to the reduction of Fe ions to nanometer-sized Fe metal and Li_2_O, while the potential slope at 1.5–2.0 V during the first charge cycle is the delithiation reaction from the active materials. For α-Fe_2_O_3_ (Figure 4a), the initial discharge/charge capacity of the cell is 698/581 mA·h·g^−1^, suggesting a Coulombic efficiency of 83.2% at the first cycling. In the 100th cycle, decreasing discharge/charge capacities to 495/481 mA·h·g^−1^ imply that there had been a significant capacity fading due to the formation of irreversible SEI layers and polarization of electrodes in the early cycles. Upon cycling, charge capacities are increased and stabilized up to 903 mA·h·g^−1^ at 700 cycles. Increasing capacity in iron-oxide-based anode materials have been observed and attributed to a gradually increasing number of active sites for the lithiation process and the stable formation of SEI layers [9,36,37,38]. In Figure 4b, the Fe_3_O_4_/C composite electrodes exhibits a discharge/charge capacity of 819/720 mA·h·g^−1^ with the Coulombic efficiency of 87.9% at the initial cycling and then gradually increasing discharge/charge capacity up to 1375/1365 mA·h·g^−1^ at the 700th cycle. In addition, the voltage profiles of the initial and 100th cycles highlight the reversibility of the Fe_3_O_4_/C composite electrodes at the early charge/discharge cycles, since there is no apparent capacity decrease but rather, gradually increasing capacities. This indicates that the Fe_3_O_4_/C composite exhibits growing active sites and stable formation of SEI layers over the charge/discharge cycles. Figure 4c further compares the cycling performance of the Fe_3_O_4_/C composite and α-Fe_2_O_3_-based electrodes up to 700 cycles at 1 A·g^−1^. The capacity of the α-Fe_2_O_3_ electrodes significantly drops at the beginning of the cycles and exhibits 395 mA·h·g^−1^ of discharge capacity at the 50th cycle, as indicated by the charge/discharge profiles. Notably, the discharge capacity of the Fe_3_O_4_/C composite electrodes decreases only up to the 7th cycle, by ~3% of initial capacity. Thus, the enhanced electrochemical performance of the Fe_3_O_4_/C composite electrodes can be attributed to the microalgae-driven carbon matrices with the unique porous and hollow structural features. Due to the synergetic contribution of these features to the buffering of volume change during the lithiation/delithiation process, there is no apparent capacity decrease in the composite microspheres but instead, a stable and long-lasting electrochemical reaction between Fe_3_O_4_/C and the electrolytes.

In Figure 4d, the rate capability results demonstrate the superior electrochemical performance of the Fe_3_O_4_/C composite electrodes for fast charge/discharge cycling. Following 10 cycles at the given current densities, the Fe_3_O_4_/C composite electrodes exhibited a higher recovery of discharge capacity than did α-Fe_2_O_3_. The improved rate capability of the Fe_3_O_4_/C composite electrodes can be attributed to the naturally doped P and S atoms with conductive carbon matrices for fast Li ion and electron transportation. In particular, P and S atoms can interact with Li ion because of their electronegativity, facilitating the Li ion transportation [22]. It has been demonstrated that carbon networks with heteroatoms (B, N, P, and S) exhibit improved performance relative to undoped carbons in LIBs [39,40]. For example, N- or B-doped graphene showed a higher rate capability than did pristine graphene at a fast charge/discharge rate [41].

**Figure 5:**
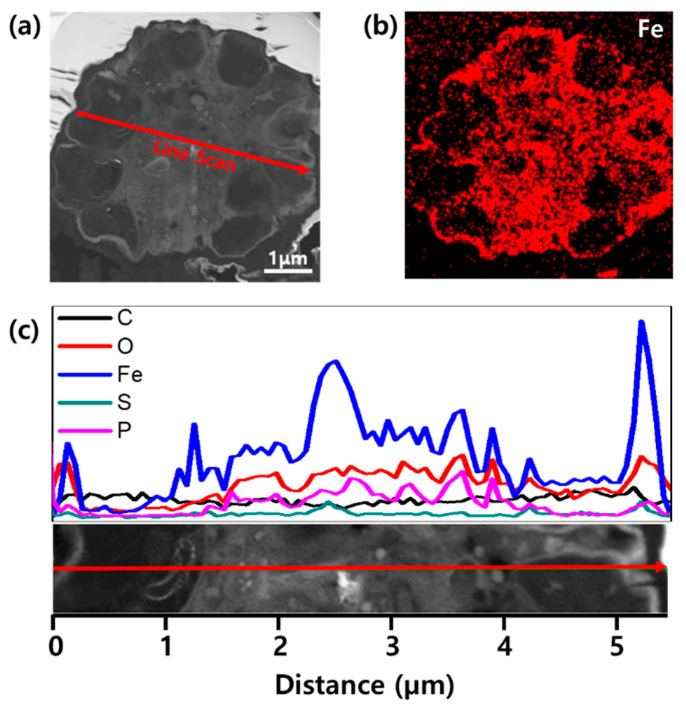
(**a**) Cross-sectional STEM image and (**b**) Fe mapping of Fe_3_O_4_/C composite microspheres after 50 cycles. (**c**) Elemental-composition profiles for C, O, Fe, S, and P atoms. The composition is identified along with the line shown in (**a**).

After 50 charge/discharge cycles, the cross-sectional morphologies and the elemental distributions of the Fe_3_O_4_/C composite electrodes were identified by FIB and TEM. Interestingly, Figure 5a shows that the hollow structures (the first level of pores) no longer exist in the composite and that the inner part of the microalgal cell (the second level of pores) can be differentiated from the other parts. As shown in Figure 5b, the inner part of the microalgal cell was not filled with the Fe elements, possibly due to the pre-existing additives, which had been filled during the preparation of the electrode with CMC binder, Super-P, and electrolytes. The line-scanned elemental-composition profiles in Figure 5c show that more C atoms were positioned where the Fe atoms were absent, between the scanning distances of around 0–1 µm and 4–5 µm. On the other hand, the hollow structures were occupied by the Fe elements, which are active materials (Figure 5b,c). The distributions of the O and P atoms qualitatively followed the composition profile of the Fe atoms. The volume taken up by the active materials can be explained by the gradual activation of composite microspheres and the formation of stable SEI layers, as demonstrated by the increasing discharge capacities over the course of the cycling. Furthermore, we speculate that, over the course of cycling, the inner part of the microalgal cell buffers the volume change.

## 4. Conclusions

Hierarchical porous Fe_3_O_4_/C composite microspheres were prepared as Li-ion battery anode materials by a microalgae-templated spray-drying method. The subsequent two-step annealing procedure developed electrochemically active and mesoporous Fe_3_O_4_/C composite microspheres with homogeneously doped microalgae-originated phosphorus and sulfur atoms within. The high-reversible capacity with good cyclability was attributed to the synergetic contribution of microalgal carbon matrices, porous structures, and doped ions, which together can buffer volume change and facilitate ion transport during fast charge/discharge cycling. Characterization of the composite electrodes after cycling further confirmed the structural integrity of the microalgal networks in correspondence with the superior electrochemical performance. The microalgae-templated spray-drying method shown here may provide new and promising strategies for recycling of biomass and the development of advanced structural composite materials for high-performance electrodes.

## Supplementary Materials

The following are available online at https://www.mdpi.com/2079-4991/10/10/2074/s1: Figure S1: TGA results of Fe_3_O_4_/C composite materials, measured in an air atmosphere. The weight increases up to 300 °C due to the oxidation of Fe_3_O_4_ under air. Figure S2: (a) N_2_ adsorption–desorption isotherm, (b) low pressure range of adsorption isotherm, (c) BJH pore size distribution, and (d) H-K micropore size distribution curves of Fe_3_O_4_/C. Figure S3: SEM image of spray dried iron nitrate solutions (0.16 M) without microalgae after annealing. Figure S4: XRD patterns of spray dried iron nitrate solutions (0.16 M) without microalgae after annealing.
