# Supplementary material for: Microalgae-Templated Spray Drying for Hierarchical and Porous Fe3O4/C Composite Microspheres as Li-ion Battery Anode Materials
Source: Nanomaterials (Basel). 2020 Oct 20;10(10):2074. doi: 10.3390/nano10102074 (PMC7589054; doi:10.3390/nano10102074)
Supplement: Supplementary file 1 [file nanomaterials-10-02074-s001.pdf]

nanomaterials

Supplementary Material

Microalgae-templated Spray Drying for Hierarchical and Porous Fe_3O_4/C Composite Microspheres as Li-ion Battery Anode Materials

Jinseok Park[1,†], Jungmin Kim[2,†], Dae Soo Jung[3], Isheunesu Phiri[2], Hyeon-Su Bae[2], Jinseok Hong[2], Sojin Kim[2], Young-Gi Lee[4,*], Myung-Hyun Ryou[2,*], Kyubock Lee[5,*]

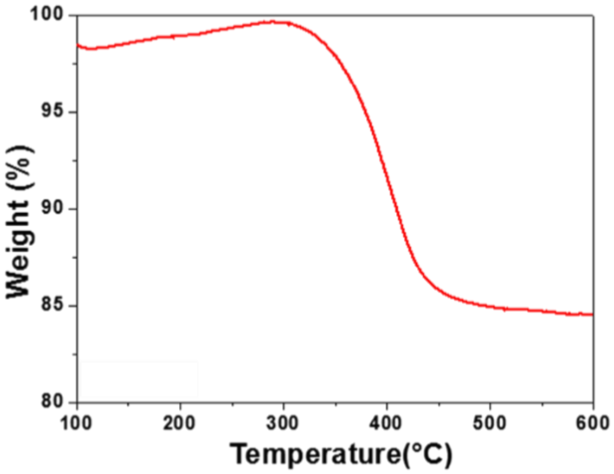

Figure 1. TGA results of Fe_3O_4/C composite materials, measured in an air atmosphere. The weight increases up to 300 °C due to the oxidation of Fe_3O_4 under air.

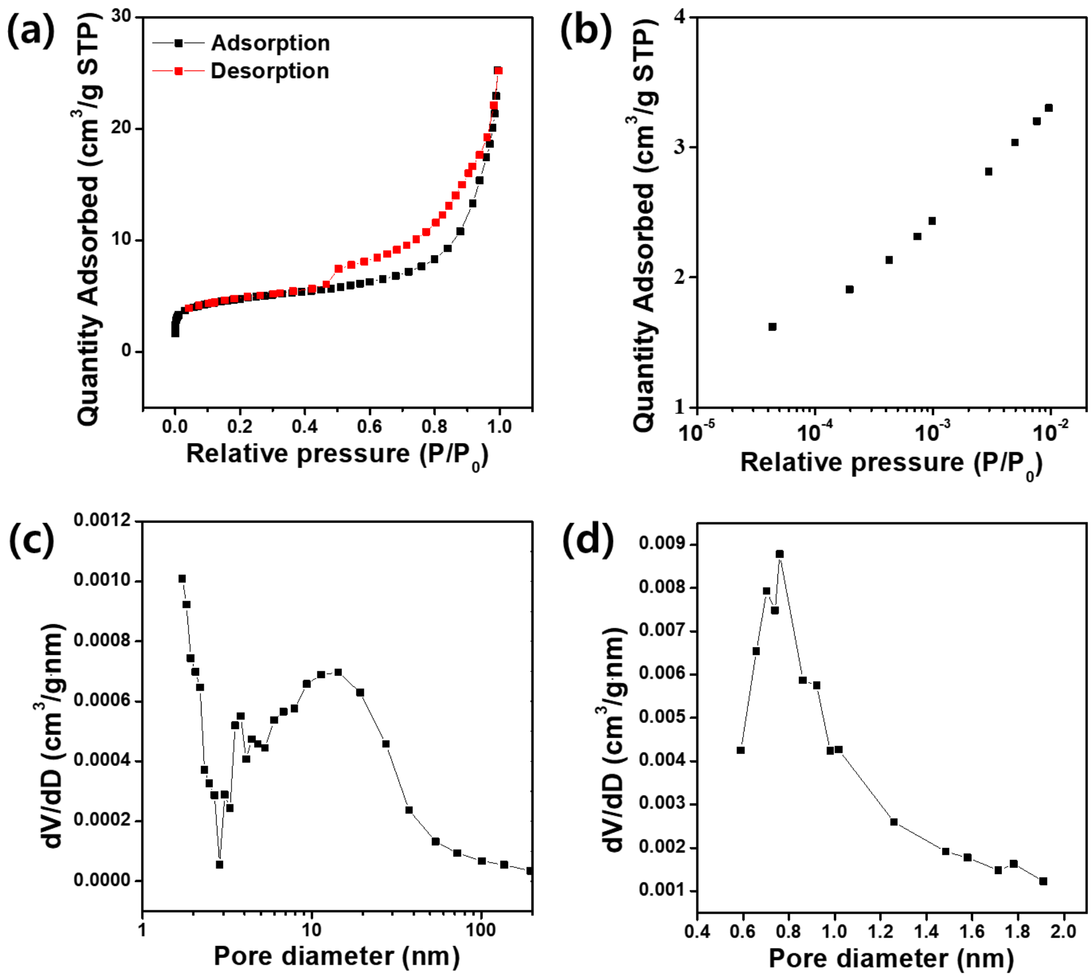

Figure 2. (**a**) N_2 adsorption–desorption isotherm, (**b**) low pressure range of adsorption isotherm, (**c**) BJH pore size distribution, and (**d**) H-K micropore size distribution curves of Fe_3O_4/C.

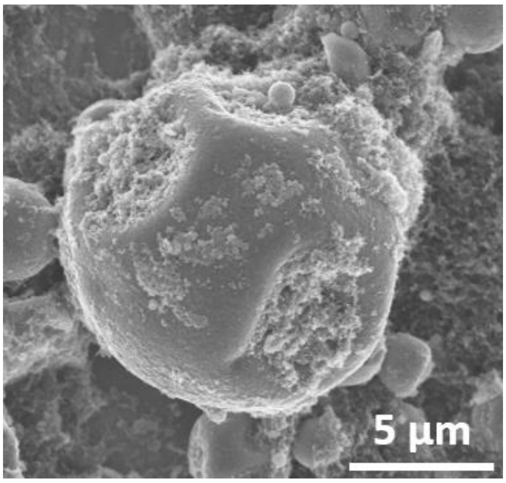

Figure 3. SEM image of spray dried iron nitrate solutions (0.16 M) without the microalgae after annealing.

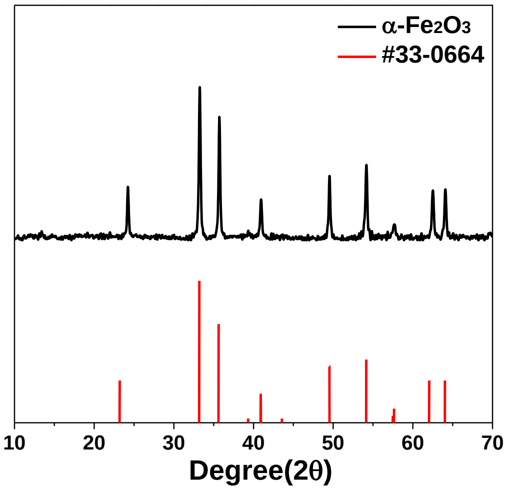

Figure 4. XRD patterns of spray dried iron nitrate solutions (0.16 M) without the microalgae after annealing.
